# Pineal cyst surgery beyond morphology: a critical evaluation of a consecutive surgical series

**DOI:** 10.1007/s00701-026-06845-z

**Published:** 2026-03-25

**Authors:** Filipe Wolff Fernandes, Assel Saryyeva, Elvis J. Hermann, Makoto Nakamura, Joachim K. Krauss

**Affiliations:** 1 Department of Neurosurgery, Hannover Medical School, Hannover, Germany; 2 Department of Neurosurgery, Academic Hospital Koeln-Merheim, University Witten/Herdecke, Cologne, Germany

**Keywords:** Pineal cyst, Microsurgery, Stereotactic drainage, Endoscopic fenestration, Ventriculocisternostomy

## Abstract

**Purpose:**

The surgical management of pineal cysts (PC) remains controversial, particularly in non-hydrocephalic patients where radiological and clinical correlations are inconsistent. Most studies rely on cyst size or morphology as surgical criteria. This study aims to assess clinical-radiological correlations and outcomes in a consecutive single-center cohort, and to compare outcomes across microsurgical, endoscopic, and stereotactic approaches.

**Methods:**

A retrospective analysis was performed on 46 symptomatic PC patients treated between 2008 and 2024. Clinical data and radiological data, including cyst dimensions, third ventricle-mesencephalic angle, aqueduct diameter, and predominant expansion were analyzed. Patients were categorized into surgical (*n* = 18) and non-surgical (*n* = 28) cohorts. Surgical outcomes were assessed using the Chicago Chiari Outcome Scale (CCOS) at 3-month, 12-month, and long-term follow-up. Statistical comparisons were conducted to correlate radiological markers with symptoms and postoperative outcomes.

**Results:**

Headache was the most common symptom in both groups (80%), followed by visual disturbances in the surgical group (33%), and vertigo in the non-surgical group (32%). Compared with the non-surgical cohort, the surgical patients had larger cysts, narrower aqueducts (0.9 vs. 1.6 mm, *p* < 0.001) and a higher prevalence of predominant anterior expansion (67% vs. 7%, *p* < 0.001). Among all operated patients, 94% achieved good or excellent CCOS outcomes at 12-months, and 93% maintained these outcomes at long-term follow-up (mean 62 months, range 16–216 months). Neither PC volume nor hydrocephalus predicted consistently postoperative outcome. Microsurgical resection (*n* = 12) achieved favorable long-term outcome (mean CCOS 14.9), but the highest complication rate (3 patients) and the highest recurrence of headache despite total PC excision. Endoscopic fenestration with ventriculostomy (*n* = 3) yielded the best long-term outcome (mean CCOS 15.7) with no recurrences. Stereotactic drainage with a catheter with a Rickham reservoir placement (*n* = 3) provided stable decompression but lower CCOS scores at long-term (mean CCOS 13.0) compared with other approaches.

**Conclusion:**

Surgery for symptomatic PC provides durable improvement when guided by radiological-clinical criteria. Aqueduct diameter was more closely associated with outcome than PC size. Microsurgical, endoscopic, and stereotactic approaches each have specific roles that should guide individualized treatment.

## Introduction

Pineal cysts (PC) are relatively common benign lesions in the pineal recess. Subsequent to the increasing use of magnetic resonance imaging (MRI), they have been described more frequently with a prevalence ranging between 1.4% and 37.5% in the general population, more common in women than in men [3, 5, 7, 43, 49]. Most PC remain unchanged in size over time or regress, but in a subset of about 5% of patients they tend to increase in size [22, 34]. Despite their noticeable prevalence, PC infrequently become symptomatic.

There is an ongoing debate about the indications for surgery with a wide range of opinions [1, 14, 18, 27, 30]. Contemporary series have demonstrated that surgical treatment of carefully selected patients with symptomatic pineal cysts can be safe and effective, with favorable functional outcomes in most cases [32]. Most series lack standardized quantification of symptoms, instead relying on categorical variables such as the presence or absence of headache or hydrocephalus, or on loosely defined subjective descriptors of improvement, which results in heterogeneous and difficult-to-compare descriptions of outcomes [11, 14, 19, 25, 27, 30, 41]. Objective clinical or radiological measures are rarely applied, and only a limited number of studies have incorporated structured outcome assessments [8, 14, 15].

Commonly accepted indications for surgery are specific symptoms due to occlusive hydrocephalus, Parinaud syndrome and visual disturbances, and pineal apoplexy [10, 44, 47, 50]. Nonetheless, there is an increased association of a variety of much more frequent symptoms, including headaches, nausea, vomiting, vertigo, sleep disorders, fatigue, paresthesias, gait problems, and endocrinopathy [3, 9, 16, 37]. The non-specificity of these symptoms in the absence of hydrocephalus challenges a clear causal association and thus the indication for surgery [3, 20, 27]. Indeed, despite a growing number of small series [12, 18, 28, 33, 38], there is no consensus concerning the indication for surgery in non-hydrocephalic PC with nonspecific symptoms [14]. According to a worldwide survey of 110 neurosurgeons, more than half considered PCs as a surgical lesion, but only 15% would indicate surgery for a PC accompanied by nonspecific symptoms [30, 31]. Surgical options for PC include microsurgical, endoscopic and stereotactic approaches, each with its pros and cons for both patient and surgeon [25, 44].

In this retrospective study, we present our experience with the surgical management of PC and aim to 1) correlate symptoms and improvement after treatment with PC morphology, and 2) compare the three different operative strategies.

## Materials and methods

### Patient selection

We identified all patients with symptomatic PC who presented at the Department of Neurosurgery, Hannover Medical School, between 2008 and 2024. Records were reviewed retrospectively, for demographic, clinical, and radiographic data, and the operative strategy. Forty-six patients with symptomatic PC were identified, including 18 who underwent surgery at our institution.

The decision to offer surgery was based on predefined clinical and radiological criteria. Surgical indications were classified as: 1) PC growth, 2) symptom-oriented, 3) suspicion of tumor, or 4) presence of hydrocephalus. Suspicion of tumor was defined by predefined MRI characteristics, including irregular or nodular contrast enhancement, wall thickening, presence of solid components, multilobulated configuration, or heterogeneous internal signal [17].

### Imaging findings and correlations

**Fig. 1:**
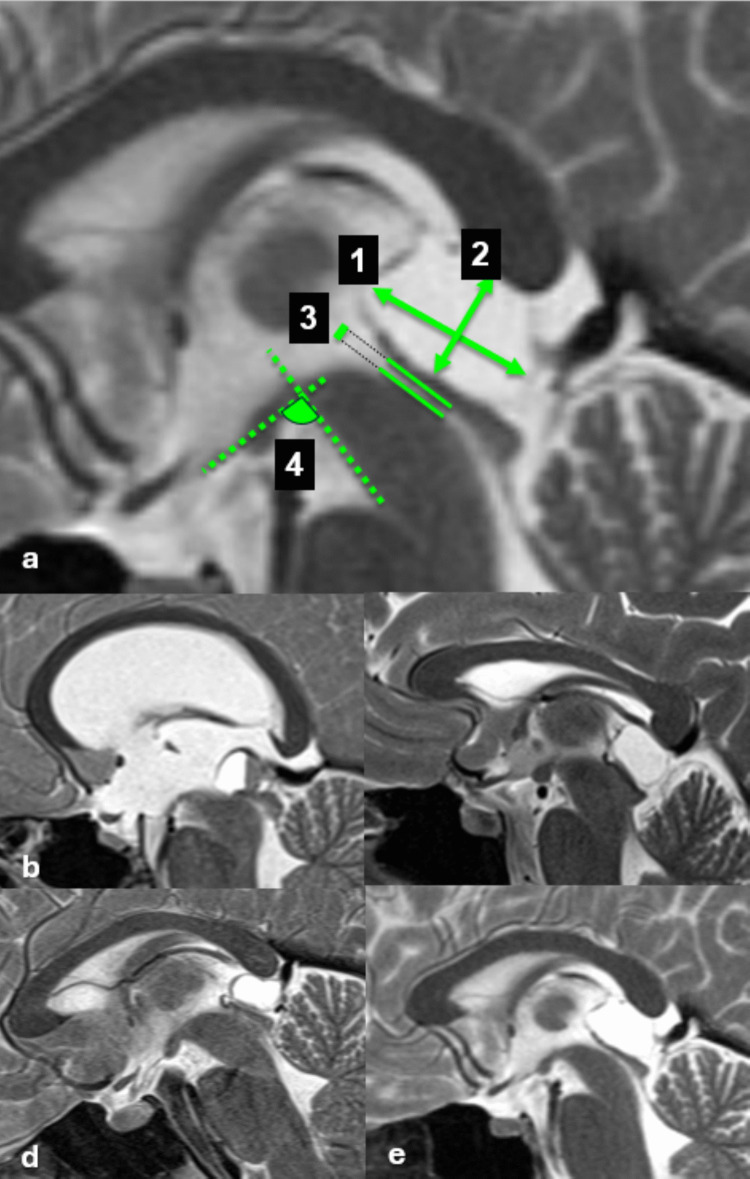
Morphometric MRI parameters in pineal cysts. Sagittal T2-weigth MRI images illustrating the radiological measurements, and examples of predominant expansions (**a**) Annotated in numbers: 1. Longitudinal cyst axis, 2. Cyst height, 3. Aqueduct diameter, 4. Third ventricle-mesencephalic angle (**b**) large cyst with anterior predominant expansion and contact/compression of the aqueduct (**c**) large cyst with inferior predominant expansion and complete obliteration of the aqueduct (**d**) large cyst with posterior predominant expansion, without significant compression of the tectum and aqueduct. **e** large cyst with superior predominant expansion and contact/compression of the splenium and intracerebral veins

All patients had preoperative magnetic resonance imaging (MRI). Radiographic measurements included: longitudinal axis and height, PC volume, predominant expansion (anterior, posterior, superior, inferior), contact/compression of adjacent structures (aqueduct, tectum, splenium, and internal cerebral veins), third ventricle-mesencephalic angle (the angle formed between the line of the floor of the third ventricle and a tangent line along the anterior border of the mesencephalon in the midsagittal plane), pineal recess crowding, as well as the presence of septations and multilobular morphology (Fig. 1). Predominant expansion was defined as the direction in which the cyst demonstrated maximal radial displacement relative to the geometric center of the pineal recess on midsagittal MRI. Anterior expansion was defined by displacement toward the third ventricle and aqueduct; posterior expansion toward the quadrigeminal cistern; superior expansion toward the splenium; and inferior expansion toward the tectal plate. Pineal recess crowding was assessed by measuring the tectum–splenium ratio on midsagittal MRI, defined as the relative distance between the tectal plate and splenium adjusted to the posterior third ventricular height, according to the method described by Eide et al. [13].

### Operative procedures

The surgical approach was determined by strategic decision-making based on patient characteristics, presence of hydrocephalus and radiological features of the PC. Surgical approaches included microsurgical resection, endoscopic fenestration and ventriculocisternostomy, and stereotactic drainage and placement of a catheter with a Rickham reservoir. Stereotactic procedures were performed by JKK and AS, endoscopic procedures by MN and EH, and open microsurgical resection by MN, EH, and JKK. All procedures were performed under general anesthesia. All patients had a postoperative CT scan on the day of surgery and were monitored overnight in the neurosurgical intensive care unit.

**Fig. 2:**
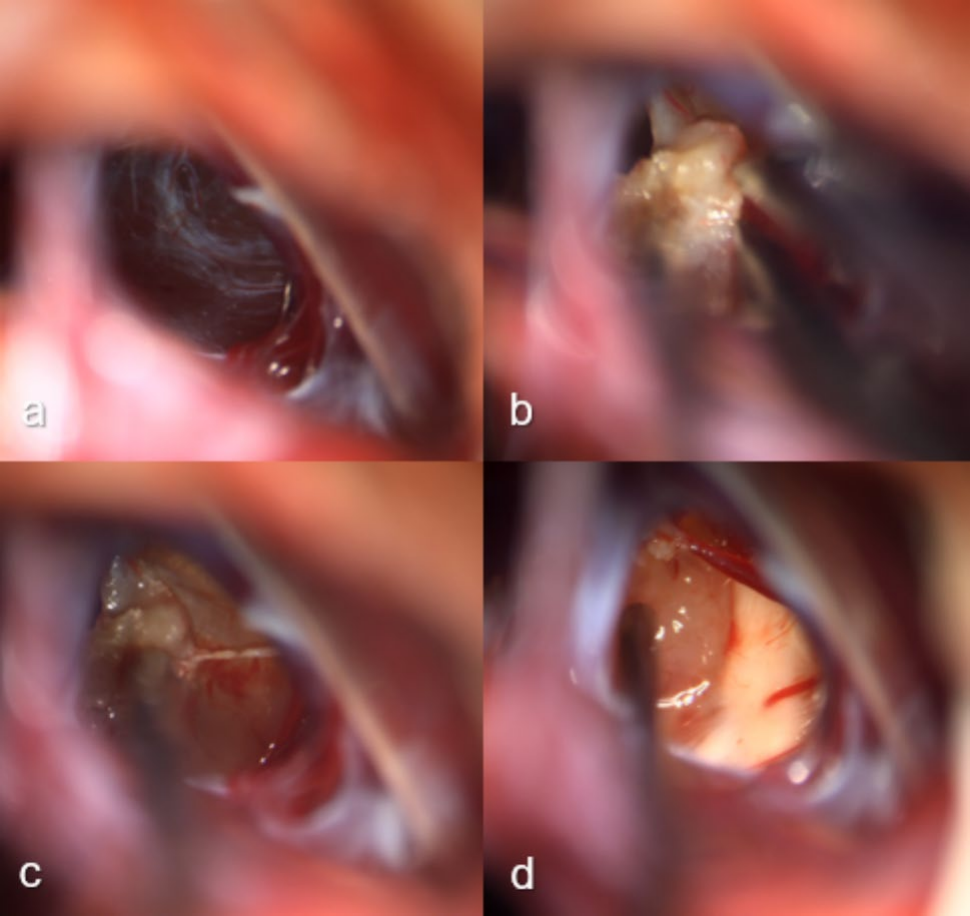
Microsurgical resection of a pineal gland cyst (18-year-old woman). **a** Initial view after the arachnoid dissection, revealing the translucent cyst wall bulging posteriorly (**b**) Sharp dissection and shrinkage of the cyst capsule with bipolar coagulation. **c** Gradual mobilization of the cyst wall from the surrounding structures in a push–pull method, avoiding injury of the surrounding structures (**d**) Complete resection of the cyst, with exposure of the tectum
- **Endoscopic cyst fenestration** was performed via a transventricular approach in patients with concomitant hydrocephalus. The patient is in a supine position with the head on a horseshoe head holder in anteflexion so that the frontal burr hole becomes the highest point of the operating field. The trajectory through the foramen of Monro and the third ventricle is guided by electromagnetic navigation as described elsewhere [23]. After visualization of the PC its wall is opened, and an aspiration catheter is inserted into the cyst, and its fluid is retracted. Gradually, the PC collapses, and additional shrinkage of the capsule is obtained by bipolar coagulation. Flaccid parts can be removed using a grasping forceps. Finally, the decompressed cerebral aqueduct is visualized as an indicator of a successful procedure reestablishing the CSF flow. The procedure is supplemented by a ventriculocisternostomy.
- **Stereotactic insertion of a catheter with a Rickham reservoir** was performed with a Riechert-Mundinger stereotactic frame with a Zamorano-Duchovny semi-arc. Planning is performed based on CT with 1.25-mm slices fused to a preoperative MRI on a workstation. Via a parietal burr hole, a cannula is inserted into the PC under stereotactic guidance which is then replaced by a catheter. Through the catheter, a small amount of contrast is injected under fluoroscopic control. The placement is accepted when the PC is fully enhanced. The contrast medium is aspirated, and the PC is flushed by saline solution. Finally, the cyst content is aspirated, and the catheter is connected to a Rickham reservoir.

- **Microsurgical resection** was performed via a midline infratentorial supracerebellar approach in the semi-sitting position as described previously [2, 24, 42]. Preoperatively, transesophageal echocardiography is performed to rule out an open foramen ovale. Through a midline skin incision, an osteoplastic suboccipital midline craniotomy is performed. The transverse sinus is identified and a midline infratentorial supracerebellar access to the pineal region is provided after Y-shaped incision of the dura. The cerebellum is retracted gently caudally after CSF release preserving the bridging veins between the surface of the cerebellum and the tentorium (Fig. 2). Arachnoid adhesions are carefully divided. The vein of Galen and the veins of Rosenthal are identified. The thickened and opaque arachnoid membranes over the quadrigeminal cistern are opened using microdissection, exposing the PC. First, the lateral margins are displayed, and the PC is slowly freed and opened. The posterior cyst wall is dissected carefully from the quadrigeminal plate and fenestrated centrally, followed by resection. After hemostasis is achieved, the dura is sutured in a watertight fashion and the bone flap is repositioned and fixated with titanium miniplates. The incision is closed in layers.

### Postoperative evaluation

The first clinical evaluation was obtained directly postoperatively, and subsequently at 3 and 12 months during regularly scheduled follow-up visits. Symptoms were assessed categorically, and functional outcomes were evaluated using the Chicago Chiari Outcome Scale (CCOS). This scale consists of four domains – pain, non-pain symptoms, functionality, and complications – each scored from 1 to 4, yielding a total score between 4 and 16. Higher scores indicate better outcomes, with scores ≥ 12 classified as good or excellent [6]. Although originally validated for Chiari malformation type I, the CCOS was selected due to its structured multidimensional assessment of pain, non-pain symptoms, functional capacity, and complications. In the absence of a disease-specific validated outcome scale for PC, it provides a reproducible framework to quantify functional outcomes beyond categorical symptom reporting. Additionally, patients were asked if there was a significant improvement in their quality of life after surgery. Radiological follow-up included a postoperative MRI obtained at least 3 months after surgery, evaluating the parameters outlined above.

#### Statistical analysis

Statistical evaluation was performed using SPSS version 21. Continuous variables were tested for normality using the Shapiro–Wilk test. Depending on data distribution, Student’s t-test or the Mann–Whitney U test was used for comparisons between two independent groups. Categorical variables were analyzed using the Chi-square test or Fisher’s exact test, as appropriate. Spearman’s rank correlation coefficient was used to assess associations between continuous variables and outcome measures. A *p*-value < 0.05 was considered statistically significant.

## Results

### Patient characteristics

Demographics data and patients’ symptoms are presented in Table 1. Surgical patients (*n* = 18) were significantly younger (mean age 21 years, range 9–33 years) compared to the non-surgical cases (*n* = 28) (mean age 35 years, range 9–76 years) (*p* = 0.006). Among the surgical patients, 12 were women, and 6 were men. Seven patients were younger than 18 years in the surgical group (39%). Headache was the most common symptom in both groups. In the surgical group, the second most common symptom was visual disturbances (33%), followed by vertigo (22%), and nausea (17%), endocrine disturbances (11%), and tinnitus (6%). Other infrequent symptoms included gait abnormalities, speech disturbances, and hallucinations. Hydrocephalus was present in 5 patients (28%). In the non-surgical group, the second most common symptom was vertigo (32%), followed by visual disturbances (7%), nausea (7%), and tinnitus (4%).

**Table 1:** Demographic data, patients’ symptoms, and indications for surgery

Group	Surgical (*n* = 18)	Non-surgical (*n* = 28)	*p*-value
Mean age	21	35	0.006
Women/Men	12/6	24/4	0.126
Headache	15 (83%)	22 (79%)	0.368
Vertigo	4 (22%)	9 (32%)	0.754
Nausea	3 (17%)	2 (7%)	0.138
Visual disturbances	6 (33%)	2 (7%)	0.008
Tinnitus	1 (6%)	1 (4%)	0.747
Endocrine disturbances	2 (11%)	0	0.148
Hydrocephalus	5 (28%)	0%	0.003
Indication for surgery			
- Symptom-oriented	9 (50%)		
- Cyst growth	2 (11%)		
- Suspected tumor	6 (33%)		
- Hydrocephalus	5 (28%)		

The indication for surgery was symptom-oriented treatment in 9 patients (50%), suspicion of tumor in 6 (33%), growth of the PC in 2 (11%), and hydrocephalus in 5 (28%). Twelve patients (66%) underwent microsurgical resection, 3 underwent endoscopic fenestration with ventriculocisternostomy (17%), and 3 underwent stereotactic drainage with a catheter with a Rickham reservoir placement (17%).

### Imaging findings and morphology

Imaging findings and morphology of the surgical and non-surgical groups are presented in Table 2. The mean longitudinal cyst axis was significantly larger in the surgical group compared to the non-surgical group (18.4 vs. 13.8 mm, *p* = 0.005), as well as the cyst height (13.1 vs. 9.1 mm, *p* = 0.007), and cyst volume (2057.0 vs. 639.9 mm^3^, *p* = 0.002). There was no difference in the third ventricle-mesencephalic angle between both groups. The surgical group had a significantly smaller aqueduct diameter compared to the non-surgical group (0.9 vs. 1.6 mm, *p* < 0.001).

**Table 2:** Preoperative morphometric characteristics of pineal cysts

Measurement	Surgical (*n* = 18)	Non-surgical (*n* = 28)	Total (*n* = 46)	*p*-value
Longitudinal cyst axis (mm)	18.4 (6.0–31.6)	13.8 (2.8–76.0)	15.6 (2.8–76.0)	0.005
Cyst height (mm)	13.1 (4.0–27.4)	9.1 (2.2–38.0)	10.7 (2.2–38.0)	0.007
Cyst volume (mm^3^)	2057.0 (87.6–7738.0)	639.9 (50.0–2830.0)	1219.6 (50.0–7738.6)	0.002
Third ventricle-mesencephalic angle (degrees)	83.2 (53.9–104.6)	85.6 (60.2–114.1)	84.7 (53.9–114.1)	0.242
Aqueduct diameter (mm)	0.9 (0–1.5)	1.6 (0.4–2.6)	1.4 (0–2.6)	< 0.001
Crowding at pineal recess	0.82 (0.34–1.0)	0.73 (0.39–0.98)	0.77 (0.34–1.0)	0.091
Septations	5 (28%)	11 (39%)	16 (34.8%)	0.424
Multilobulated cysts	2 (11%)	7 (25%)	9 (19.6%)	0.448
Predominant expansion				
Anterior	12 (67%)	2 (7%)	14 (30.4%)	< 0.001
Posterior	10 (56%)	24 (86%)	34 (73.9%)	0.023
Inferior	3 (17%)	15 (54%)	18 (39.1%)	0.012
Superior	6 (33%)	13 (46%)	19 (41.3%)	0.379
Contact/compression to key structures				
Aqueduct	10 (56%)	5 (18%)	15 (32.6%)	0.008
Tectum	12 (67%)	17 (61%)	29 (63.0%)	0.683
Splenium	9 (50%)	10 (36%)	19 (41.3%)	0.337
Intracerebral veins	8 (44%)	3 (11%)	11 (23.9%)	0.009

In the surgical group, the predominant expansion was significantly more frequent in the predominant anterior expansion (67% vs. 7%, *p* < 0.001), while predominant posterior and inferior expansions were more prevalent in the non-surgical group (86% vs. 56%, *p* = 0.023; 54% vs. 17%,  = 0.012, respectively). Contact/compression with the aqueduct was significantly more frequent in the surgical group (56% vs. 18%, *p* = 0.008), as well as the contact/compression with the intracerebral veins (44% vs. 11%, *p* = 0.009). The frequency of septations and multilobulated cysts did not differ between groups.

### Symptom—imaging correlations

Headaches were more frequently present in PC with a longer longitudinal axis (16.3 vs. 12.2 mm) and height (11.1 vs. 8.7 mm), as well as a larger volume (1346.9 vs. 646.7 mm^3^), but this lacked statistical significance. The presence of vertigo was associated with an inferior predominant expansion (*p* = 0.033). Pineal recess crowding, assessed by the tectum–splenium ratio, was significantly higher in patients presenting with nausea compared to those without nausea (0.94 vs. 0.74, *p* = 0.002). There was no other significant correlation with symptoms.

Hydrocephalus was significantly associated with contact/compression of the aqueduct (*p* = 0.033) and predominant anterior expansion (*p* = 0.001), while the non-hydrocephalic cases were associated with a predominant posterior expansion (*p* = 0.013). The presence of hydrocephalus was associated with a smaller aqueduct diameter (0.6 vs. 1.4 mm, *p* = 0.016).

In the surgical group, the preoperative longitudinal cyst axis was significantly longer in patients presenting with headaches (19.5 vs. 9.6 mm, *p* = 0.026) compared to patients without headaches, while such differences were not observed in cyst height, volume, aqueduct diameter, or third ventricle-mesencephalic angle. Visual disturbances were associated with a smaller cyst volume (1451.9 vs. 2442.1 mm^3^, *p* = 0.035). No other symptoms correlated significantly with morphological characteristics or contact/compression of structures.

### Postoperative clinical and imaging outcome

All patients were available for 3-month follow-up, 17 for 12-month follow-up (94%), and 15 patients for long-term follow-up (83%) (mean 62 months, range 16–216 months). Headaches improved in all patients at 3-month follow-up (50% completely, 50% partially), but recurrence of headache occurred in 5 patients on long-term follow-up (31%). Visual disturbances resolved in all cases, with only one case of recurrence (12%). Vertigo improved in 4 out of 6 cases (67%) with one recurrence at 12-month follow-up (17%). Symptom improvement was documented in 78%, 94% and 89% at 3-month, 12-month and long-term follow-up, respectively.

Considering functional outcome, 94% of patients achieved good or excellent results (CCOS ≥ 12) at 3-month follow-up, which was maintained at 12-month follow-up. Sustained benefit was observed in 14 out of 15 patients at long-term follow-up (93%). None of the cases with recurrence of symptoms presented with PC regrowth. We did not identify any prognostic factor associated with either clinical or imaging outcome.

Patients with a greater cyst height preoperatively showed more frequent improvement of headache compared to those with only partial relief (mean 17.3 mm vs. 10.8 mm, *p* = 0.038). A narrower aqueduct diameter preoperatively was significantly associated with higher CCOS scores at 3-month follow-up (*p* = 0.041), but this association did not persist at 12 months or long-term follow-up. At 12-month follow-up, patients with preoperative tectal contact/compression had significantly lower CCOS scores compared to those without contact/compression (mean score 13.9 vs. 15.6, *p* = 0.004). No other imaging findings were correlated with symptom improvement or CCOS outcomes.

### Outcome by surgical technique

An overview of the functional outcomes of patients according to the CCOS scores with respect to the different surgical techniques is shown in Table 3. Microsurgical resection was used in all patients with a suspicion of a pineal tumor to obtain a definitive histological diagnosis. Complete headache improvement was achieved in 4 out of 10 patients (40%), and partial improvement in 6 (60%), while recurrence of headaches occurred in 3 patients (27%). Satisfactory outcome occurred in 9 patients (75%), 11 patients (100%), and 9 patients (100%) at 3-month, 12-month, and long-term follow-up, respectively. Major intra- or postoperative complications occurred in 3 patients (25%) in the microsurgical group, including one case with cerebellar hemorrhage requiring surgical evacuation and placement of an external ventricular drain, and later a ventriculoperitoneal shunt; one case with a supratentorial subdural hematoma requiring evacuation; and one case of cerebellar swelling necessitating decompressive surgery.

**Table 3:** Chicago Chiari outcome scale (CCOS) and complications by surgical technique

Procedure	Microsurgical (12)	Endoscopic (3)	Stereotactic (3)
3-month FU	13.3 (6–16)	15 (14–16)	13.7 (12–15)
12-month FU	14.2 (13–16)	15.3 (14–16)	14.3 (14–15)
Long-term FU	14.9 (13–16)	15.7 (15–16)	13 (10–15)
Complications	3	0	0

In the endoscopic group, headache, vertigo, and visual disturbances improved in all patients, and one recurrence of headache has been observed (50%). No complications were found. Satisfactory outcome has been achieved in all patients at 3-month, 12-month, and long-term follow-up. Also, in comparison to the other groups, the highest CCOS scores were achieved in all follow-up periods.

In the stereotactic group which consisted of patients with deep-seated lesions with predominant inferior expansion, 2 patients reported partial headache relief, whereas one patient improved significantly. Vertigo improved in two patients and recurred in one patient. Visual disturbances improved in 2 patients. In no case, aspiration of fluid via the Rickham reservoir became necessary. Satisfactory outcome was achieved in all patients at 12-month follow-up, and in 2 patients (67%) at both 3-month and long-term follow-up.

**Fig. 3:**
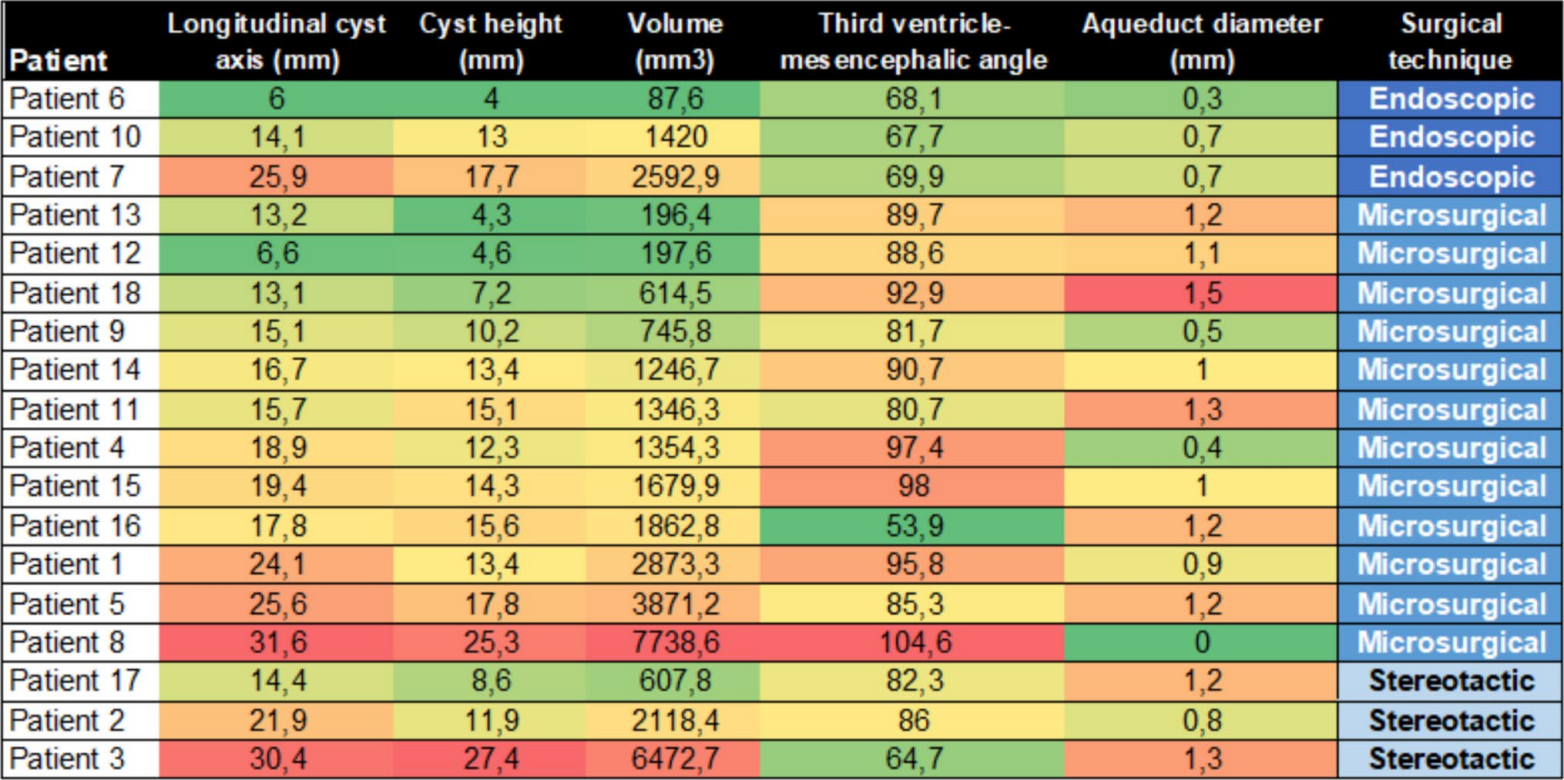
Morphological measurements of the pineal cyst and the third ventricle-mesencephalic angle. The heatmap displays the cyst dimensions and the third ventricle-mesencephalic angle according to the type of surgical technique. Red indicates higher values and green indicates smaller values

PC dimensions, third ventricle-mesencephalic angle and aqueduct diameter for the single patients undergoing different surgical techniques are shown in a heatmap in Fig. 3. There was no statistically significant difference in CCOS or symptom improvement between surgical groups, although the endoscopic group showed the most consistent higher functional outcomes, followed by the microsurgical resection group, while the stereotactic group had the shortest hospital stay.

### Pediatric subgroup

Ten patients were younger than 18 years at presentation (mean age 13 years, range 9–17 years). Seven patients underwent surgical treatment, including four microsurgical resections, two endoscopic fenestrations with ventriculocisternostomy, and one stereotactic drainage with placement of a catheter and a Rickham reservoir. Three patients were managed non-surgically.

Headache was the most common symptom (8/10), followed by vertigo (7/10), nausea (2/10), and visual disturbances (2/10). Hydrocephalus was present in three patients. The mean longitudinal cyst axis was 23.2 mm (range 6.0–76.0 mm), the mean cyst height was 14.3 mm (range 4.0–38.0 mm), and the mean cyst volume was 1936.6 mm^3^ (range 87.6–7738.6 mm^3^). The mean aqueduct diameter was 0.3 mm (range 0–1.0 mm). Contact/compression of adjacent structures included aqueduct compression in three patients, tectal contact/compression in five, splenial contact/compression in three, and contact/compression of the internal cerebral veins in four patients. Septations of the PC were present in one patient. Predominant posterior expansion was observed in 6 cases, anterior expansion in 5, inferior expansion in 3, and superior expansion in 3 cases.

All surgically treated pediatric patients achieved good or excellent functional outcomes (CCOS ≥ 12) at 3-month and 12-month follow-up. The 6 out of 7 patients who were available for long-term follow-up (mean 72 months, range 24–146 months), sustained good or excellent outcome.

## Discussion

This study shows that diagnostic and therapeutic decisions in pineal cysts are complex, particularly in patients without hydrocephalus. Previous studies focused mainly on cyst dimensions, while our data also takes into account that the predominant expansion of the cyst might be related. In particular, aqueduct narrowing was associated with better postoperative outcomes. These results support patient selection based also on morphological patterns and clinical presentation rather than on size alone.

The pathogenesis and natural history of PC remain poorly understood. Proposed hypotheses include enlargement of the embryonic pineal recess, ischemic degeneration, or hemorrhagic transformation of the parenchyma, as well as additional hormonal influences, given their preponderance in women [3, 4, 9]. Longitudinal studies indicated that most PC remain stable or may even regress, with only a 5% mean risk of growth, complicating surveillance and decision-making for intervention based solely on growth [5, 22, 48]. Surgery should be guided not by PC size, but by the clinical picture and its impact on quality of life. Also, one cannot discard the importance of ruling out a pineal neoplasm, particularly when atypical features are observed (septations, thicker wall, multilobulated shape, multicystic appearance) [17, 21, 22].

Symptoms attributed to PC are often nonspecific [36]. Headaches, the most frequent complaint, often holocephalic and intermittent, have no consistent correlation with imaging [1, 19]. Seifert et al. found an increased prevalence of migraine and tension-type headache in patients with PC [45], while a recent cohort of 154 patients with PC showed no correlation with the presence and evolution of headache [26]. A meta-analysis confirmed that non-migraine headaches respond better to surgery than migraine headaches, whose response is variable [33]. In our cohort, larger PC showed a higher prevalence of headache, which first would suggest a direct size-symptom correlation. However, the variable improvement rates after microsurgical resection and the recurrence of headache indicate that the mass effect is not the sole mechanism for the occurrence of headache. This underscores the need for accurate preoperative evaluation and realistic counseling.

Other symptoms, such as visual disturbances, vertigo, nausea and cognitive impairment showed no correlation with size in the literature [1, 19, 38]. In our series, visual disturbances were associated with smaller PC, while vertigo was linked to predominant inferior expansion, suggesting an interference with tectal function rather than direct contact/compression [33].

Several mechanisms for symptomatic PC have been proposed, but their causality remains debated. Our findings support a dominant role for cerebrospinal fluid (CSF) pathway compromise. Predominant anterior expansion and reduced aqueduct diameter were more frequent in the surgical group, suggesting intermittent obstruction as a key factor [16, 19]. Consistent with prior findings of narrower rostral aqueduct diameters in symptomatic patients compared to controls [41], narrower aqueducts predicted better functional outcomes, emphasizing the role of aqueduct diameter measurement in identifying patients at risk of intermittent obstruction and hydrocephalus. Operated patients frequently showed contact/compression of intracerebral veins, particularly in non-hydrocephalic patients, which may lead to venous congestion and impaired CSF pulsatility [12, 14]. Additionally, patients with larger PC height demonstrated greater headache improvement, supporting the role of pineal recess crowding in the generation of headaches [49]. Melatonin dysregulation remains a plausible secondary contributor to headache, which could explain the variable outcome after surgical treatment [39, 40].

Our surgical results demonstrate that carefully selected patients guided by radiological-clinical correlations can achieve sustained improvement, both with and without hydrocephalus. Good or excellent CCOS scores were reached in 94% at 12 months and maintained in 93% at long-term follow-up, underscoring a durable surgical benefit over an average of more than 4 years. These rates compare favorably with the literature, including a meta-analysis of 280 patients reporting 47–96% improvement rates [33] and recent single-center series describing 89–93% favorable outcomes [18, 25]. Headache recurrence was common but did not significantly affect overall functional outcome.

Each surgical technique was applied to a distinct subset of patients with identifiable clinical and morphologic features, delineating its specific indications and role. Microsurgical resection enables complete excision and definitive histological diagnosis and is therefore preferred in cases with atypical imaging features or suspected neoplasm. However, it requires a supracerebellar infratentorial approach and was associated with the highest complication rate in our series (25%) as well as the greatest risk of headache recurrence despite cyst removal. Endoscopic fenestration combined with ventriculostomy offers a minimally invasive option, particularly in patients with hydrocephalus or predominant anterior expansion compromising CSF pathways. In our cohort, this strategy yielded the best functional outcomes and no recurrences during follow-up. Although cyst regrowth after fenestration has been reported [11, 18, 37], the additional ventriculostomy may contribute to durable symptom control, as it also has been described for other pineal region lesions [35]. Stereotactic drainage with catheter and reservoir placement represents the least invasive alternative and may be considered in deep-seated or high-risk cases. While stable decompression was achieved, functional outcomes were less favorable compared with microsurgical and endoscopic approaches. Despite earlier reports of more frequent recurrences after aspiration alone [29, 46], catheter implantation with a Rickham reservoir remains a practical option in selected patients.

Taken together, our findings support an individualized treatment algorithm: microsurgery for atypical or suspicious lesions of the pineal gland, endoscopy for hydrocephalus and predominant anterior expansion, and stereotactic drainage for deep-seated lesions, or high-risk cases. Beyond surgical selection, aqueduct diameter and predominant expansion are meaningful radiological markers that help identify surgical candidates more accurately than PC size alone. The use of structured outcome scales such as the CCOS in patients with PC [18] adds value by quantifying functional outcomes rather than merely symptom reduction, addressing prior limitations of categorical symptom reporting [12, 14, 33].

Limitations of our study include its retrospective design, lack of a conservatively managed control group with follow-up data, heterogeneous radiological data due to in-hospital and external imaging, and reliance on subjective symptom reporting. The potential contribution of placebo effects or spontaneous symptom resolution cannot be excluded. Future prospective multicenter studies using objective outcome metrics are needed to confirm these findings.

## Conclusion

This series supports the view that surgery for PC can provide durable benefit even without hydrocephalus when guided by clinical-radiological criteria. Aqueduct diameter appears to be a more reliable predictor of outcome than PC size. Microsurgical, endoscopic, and stereotactic approaches each have roles depending on the anatomical and clinical context.

## Data Availability

No datasets were generated or analysed during the current study.
